# Tracking cell turnover in human brain using ^15^N-thymidine imaging mass spectrometry

**DOI:** 10.3389/fnins.2023.1274607

**Published:** 2023-09-28

**Authors:** Sebastian S. Roeder, Elisa A. Bonnin, Ting-Di Wu, Jean-Luc Guerquin-Kern, Samir Jabari, Sebastian Brandner, Ilker Y. Eyüpoglu, Stephanie Gollwitzer, Hajo M. Hamer, Stefan T. Gerner, Thorsten R. Doeppner, Christoph Rummel, Elisabet Englund, Ralph Heimke-Brinck, Tobias Borst, Christoph Daniel, Kerstin Amann, Ursula Schlötzer-Schrehardt, Anton B. Tonchev, Karl Roessler, Stefan Schwab, Olaf Bergmann, Silvio O. Rizzoli, Hagen B. Huttner

**Affiliations:** ^1^Department of Neurology, University of Erlangen-Nuremberg, Erlangen, Germany; ^2^Center for Regenerative Therapies, Technische Universität Dresden, Dresden, Germany; ^3^Department of Neuro- and Sensory Physiology, University Medical Center Göttingen, Excellence Cluster Multiscale Bioimaging, Center for Biostructural Imaging of Neurodegeneration, Göttingen, Germany; ^4^Institut Curie, PSL University, Université Paris-Saclay, CNRS UAR2016, Inserm US-43, Multimodal Imaging Center, Paris, France; ^5^Department of Neuropathology, University of Erlangen-Nuremberg, Erlangen, Germany; ^6^Department of Neurosurgery, University of Erlangen-Nuremberg, Erlangen, Germany; ^7^Department of Neurosurgery, Technische Universität Dresden, Dresden, Germany; ^8^Department of Neurology, Justus Liebig University, Gießen, Germany; ^9^Department of Veterinary Physiology and Biochemistry, Justus Liebig University, Gießen, Germany; ^10^Department of Clinical Sciences, University of Lund, Lund, Sweden,; ^11^Pharmacy, University of Erlangen-Nuremberg, Erlangen, Germany; ^12^Department of Nephropathology, Institute of Pathology, Friedrich-Alexander-University Erlangen-Nuremberg, Erlangen, Germany; ^13^Department of Ophthalmology, University Hospital, Friedrich-Alexander-University Erlangen-Nuremberg, Erlangen, Germany; ^14^Department of Anatomy and Cell Biology and Stem Cell Biology Research Institute, Medical University Varna, Varna, Bulgaria; ^15^Department of Neurosurgery, University of Vienna, Vienna, Austria; ^16^Department of Cell and Molecular Biology, Karolinska Institute, Stockholm, Sweden; ^17^Institute of Pharmacology and Toxicology, Universitätsmedizin Göttingen, Göttingen, Germany

**Keywords:** neurogenesis, adult, hippocampus, cell turnover, glioblastoma, Nano-SIMS, DNA-labeling

## Abstract

Microcephaly is often caused by an impairment of the generation of neurons in the brain, a process referred to as neurogenesis. While most neurogenesis in mammals occurs during brain development, it thought to continue to take place through adulthood in selected regions of the mammalian brain, notably the hippocampus. However, the generality of neurogenesis in the adult brain has been controversial. While studies in mice and rats have provided compelling evidence for neurogenesis occurring in the adult rodent hippocampus, the lack of applicability in humans of key methods to demonstrate neurogenesis has led to an intense debate about the existence and, in particular, the magnitude of neurogenesis in the adult human brain. Here, we demonstrate the applicability of a powerful method to address this debate, that is, the *in vivo* labeling of adult human patients with ^15^N-thymidine, a non-hazardous form of thymidine, an approach without any clinical harm or ethical concerns. ^15^N-thymidine incorporation into newly synthesized DNA of specific cells was quantified at the single-cell level with subcellular resolution by Multiple-isotype imaging mass spectrometry (MIMS) of brain tissue resected for medical reasons. Two adult human patients, a glioblastoma patient and a patient with drug-refractory right temporal lobe epilepsy, were infused for 24 h with ^15^N-thymidine. Detection of ^15^N-positive leukocyte nuclei in blood samples from these patients confirmed previous findings by others and demonstrated the appropriateness of this approach to search for the generation of new cells in the adult human brain. ^15^N-positive neural cells were easily identified in the glioblastoma tissue sample, and the range of the ^15^N signal suggested that cells that underwent S-phase fully or partially during the 24 h *in vivo* labeling period, as well as cells generated therefrom, were detected. In contrast, within the hippocampus tissue resected from the epilepsy patient, none of the 2,000 dentate gyrus neurons analyzed was positive for ^15^N-thymidine uptake, consistent with the notion that the rate of neurogenesis in the adult human hippocampus is rather low. Of note, the likelihood of detecting neurogenesis was reduced because of (i) the low number of cells analyzed, (ii) the fact that hippocampal tissue was explored that may have had reduced neurogenesis due to epilepsy, and (iii) the labeling period of 24 h which may have been too short to capture quiescent neural stem cells. Yet, overall, our approach to enrich NeuN-labeled neuronal nuclei by FACS prior to MIMS analysis provides a promising strategy to quantify even low rates of neurogenesis in the adult human hippocampus after *in vivo*^15^N-thymidine infusion. From a general point of view and regarding future perspectives, the *in vivo* labeling of humans with ^15^N-thymidine followed by MIMS analysis of brain tissue constitutes a novel approach to study mitotically active cells and their progeny in the brain, and thus allows a broad spectrum of studies of brain physiology and pathology, including microcephaly.

## 1. Introduction

Studying the generation of new cells in the human brain is of fundamental importance, for at least two reasons. First, with regard to brain development, the generation of neurons is a key feature, and impairment of this process underlies various developmental disorders, notably microcephaly. Second, with regard to the maintenance of brain function, the ability to replace neurons is of pivotal relevance for aging research and regenerative medicine. As to the latter, landmark studies in mice and rats provided compelling evidence that lifelong neurogenesis occurs in distinct regions of the adult rodent brain, i.e., the subgranular zone of the hippocampus and within the subventricular zone of the lateral ventricles (Bergmann et al., 2015). These studies utilized the thymidine analog bromodeoxyuridine (BrdU) to identify newly born neurons by labeling cycling progenitor and stem cells which pass on the label to their progeny (Choi et al., 2018; Berdugo-Vega et al., 2020). In contrast to these studies conducted in animals, the magnitude of neurogenesis in the adult human brain is still intensely debated. A main reason for this debate is that much of the methodology to track stem and progenitor cells used to demonstrate neurogenesis in the adult rodent brain cannot be applied to humans (Bergmann et al., 2015).

An analysis in 1998 provided first evidence for adult neurogenesis in the human hippocampus, studying cancer patients who were treated with BrdU for diagnostic purposes (Eriksson et al., 1998). However, the low number of cancer patients and the observation of only few BrdU-labeled neurons in the adult human hippocampus have raised doubts about the general relevance of this study. A decade ago Jonas Frisen and colleagues made use of the increase in ^14^C atmospheric concentration due to the above-ground nuclear tests conducted in the 1950’s and 1960’s. Nuclear bomb-based ^14^C integration into neuronal DNA allowed to determine the age of human neurons. Combining this strategy with mathematical modeling provided strong evidence for neurogenesis in the human hippocampus as well as in the human striatum (Spalding et al., 2013; Ernst et al., 2014). Although this strategy is powerful in establishing the existence of newly generated neurons, it requires at least 1 million neurons and does not allow analysis at a single-cell level (Spalding et al., 2005a,b). Moreover, mathematical modeling is required, and difficulties remain in distinguishing between *de novo* DNA synthesis and DNA repair. Finally, this intriguing approach in future is limited by a decreasing applicability due to the “aging” of the above-ground nuclear bomb test-derived ^14^C peak.

The pioneering work of Claude Lechene and colleagues over the past decade (Steinhauser et al., 2012; Senyo et al., 2013) has provided another innovative approach to study cell turnover in tissues that overcomes these ambiguities and should allow to settle the debate regarding whether or not, and if so, to what extent, new neurons are being generated in the adult human brain. This approach involves *in vivo* labeling with ^15^N-thymidine, a non-hazardous form of thy-midine carrying the stable isotope ^15^N. Like BrdU, ^15^N-thymidine is incorporated into genomic DNA during the S-phase of the cell cycle of mitotically active stem and progenitor cells and inherited by the cells generated therefrom (Steinhauser et al., 2012; Senyo et al., 2013). A crucial feature of this approach is the subsequent analysis of the ^15^N-thymidine–labeled tissue by multiple-isotope im-aging mass spectrometry (MIMS), which allows the quantitative imaging of stable isotope labels (in this case ^15^N) in cells using a secondary ion mass spectrometer (NanoSIMS). This type of imaging yields information about the incorporation of ^15^N-thymidine into DNA at the single-cell level with subcellular resolution, in tissue sections of the organ of interest.

The approach of *in vivo* ^15^N-thymidine labeling followed by MIMS analysis has successfully been applied to non-neural human tissues such as adipose tissue, the heart and the hematopoietic system (Steinhauser et al., 2012; Senyo et al., 2013; Guillermier et al., 2017), as well as to mouse brain (Enikolopov et al., 2014). Here, we report the first application of this powerful approach to investigate cell turnover, including neurogenesis, in the adult human central nervous system, notably the brain, *in vivo*. Specifically, we subjected a human glioblastoma patient and a human patient with pharmaco-refractory focal temporal epilepsy to *in vivo* ^15^N-thymidine labeling followed by MIMS analysis of the resected tumor tissue and the hippocampus, respectively, to determine whether the generation of new neural cells can be detected with this technology. While we observed ^15^N-thymidine–labeled cells in the tumor tissue, this was not the case for the limited number of dentate gyrus cells analyzed in the hippocampal tissue. Nonetheless, our data provide proof-of-principle evidence that *in vivo* ^15^N-thymidine labeling of adult humans followed by MIMS analysis of the relevant resected brain tissue should be a suitable approach to determine, in the future, whether new neurons are being generated in the adult human brain and if so, to what extent those neurons are generated. Our approach of *in vivo* ^15^N-thymidine labeling followed by MIMS analysis should also be considered for studying disorders of brain development, for example by analyzing brain tissue from patients with microcephaly.

## 2. Methods

### 2.1. Patient recruitment and characteristics

We recruited two adult patients admitted to the Department of Neurology of the University Hospital Erlangen for participation in our study. The main inclusion criteria were scheduled neurosurgical procedure with excision of neural tissue and the ability to understand the nature of the study. Informed consent was obtained after detailed information about the study and its pioneering character. Ethical approval for the study was granted by the local institutional review board (Erlangen 195_17 B).

Patient 1 was a 66-year old female that was admitted due to a focal to bilateral motor seizure and MR imaging revealed a right-sided temporal mass (Supplementary Table 1). Radiological and clinical finding were suspicious of a higher grade glioma or other primary central-nervous tumor (Supplementary Figure 1). Surgery was scheduled for removal of the mass and histopathological diagnosis for further planning of treatment. While waiting for surgery ^15^N-thymidine infusion was administered (see below). Surgery was performed without complications and histopathological findings confirmed the diagnosis of glioblastoma multiforme (glioma WHO °IV, see Supplementary Figure 2). The patient in the following underwent combined radio-chemotherapy according to international guidelines.

Patient 2 was a 26-year old female with a diagnosis of drug-refractory right-sided focal temporal epilepsy that presented to our specialized epilepsy center for screening of suitability for epilepsy surgery, i.e., amygdalo-hippocampectomy (Supplementary Table 1). MRI imaging did not reveal pathological findings (Supplementary Figure 3). After she underwent pre-surgical evaluation for potential surgical treatment, interdisciplinary consensus recommended a combined amygdalohippocampectomy that was scheduled 7–8 weeks thereafter. While waiting for surgery ^15^N-thymidine infusion was administered 43 days prior to surgery. Surgery was performed without complications and resulted in a significant reduction of monthly seizure rate. Histopathological evaluation showed normal findings, with a questionable mild hippocampal sclerosis (Supplementary Figure 4).

### 2.2. Administration of ^15^N-thymidine

Human clinical trial grade endotoxin-free ^15^N-thymidine, specifically produced for patient infusion in the context of this pioneer study meeting all process-related regulatory requirements for human clinical trials, was obtained from Eurisotop (Saint-Aubin Cedex, France). The final infusion was prepared by the Pharmacy of the University Hospital Erlangen in accordance with good manufacturing practice guidelines. We infused a total of 250 mg ^15^N-thymidine, the same for both patients, over a 24 h period directly before the neurosurgical procedure in patient 1, and 43 days prior to surgery in patient 2. After an initial bolus of 10 mg ^15^N-thymidine, its administration was continued for the following 24 h at a rate of 10 mg/h. Both patients were monitored and underwent infusion without any complications or side effects.

### 2.3. Sample and tissue collection and preparation

Peripheral blood samples were obtained immediately before and after ^15^N-thymidine administration (postoperative day 22 in patient 1 and on day 54 post infusionem in patient 2), collected into a tube containing EDTA on ice. Samples were centrifuged for 10 min at 2,000 g and the buffy coat was collected. After lysis of red blood cells using a lysis buffer (Thermo Fisher, Germany) the samples were washed with PBS and fixed in 4% PFA/2.5% glutaraldehyde for 1 h at room temperature (RT). The cells were then pelleted in a 200 μL test tube and embedded in low-melting agarose (Biozym Scientific, Oldendorf/Germany) to facilitate further handling and storage.

CNS tissue specimens were obtained from the intraoperatively resected tissue that was not needed for histopathological workup. Immediately after resection and splitting of the tissue samples were stored in ice-cold PBS for several minutes to enable transport from the operating room to the laboratory. Here samples were fixed in 4% PFA/2.5% glutaraldehyde for 12 h at RT.

### 2.4. Microdissection of the dentate gyrus

Identification and sampling of the dentate gyrus in the hippocampus specimen was performed under a stereomicroscope (see Supplementary Figure 5), followed by dissection in serial 1 mm cubes that were investigated sequentially. After sectioning of the tissue cubes the granular cell layer was localized by light microscopy and was visualized by MIMS (Supplementary Figures 4 and 5).

### 2.5. Isolation of neuronal nuclei and flow cytometry

Neuronal nuclei were labeled and sorted using the neuronal marker NeuN. After embedding and post-processing, brain tissue was thawed and homogenized in ice-cold lysis buffer (0.32 M sucrose, 5 mM CaCl2, 3 mM magnesium acetate, 2.0 mM EDTA, 10 mM Tris–HCl [pH 8.0]) by using a glass dounce homogenizer and the homogenate suspended in an ice-cold 1.7 M sucrose solution (Huttner et al., 2014, 2018). As described previously (Spalding et al., 2013; Huttner et al., 2018), the suspension was then layered onto a cushion of 10 mL 1.7 M sucrose solution (1.7 M sucrose, 5 mM CaCl2, 3 mM magnesium acetate, 2.0 mM EDTA, 10 mM Tris–HCl [pH 8.0]) in a high-speed centrifuge tube (Beckman Centrifuge Tubes #363664) and spun at 26,500 × g for 2 h at 4°C (Beckman Avanti with JS13.1 rotor). The supernatant was discarded, and the pellet was resuspended with 1 mL of nuclei storage buffer (0.43 M [15%] sucrose, 70 mM KCl, 2 mM MgCl2, 10 mM Tris–HCl [pH 7.2]) for flow cytometry analysis (Spalding et al., 2005a; Huttner et al., 2014).

Isolated cell nuclei were incubated with primary antibody NeuN (1800, mouse, A60, Chemicon) directly conjugated to Alexa Fluor 488 (Alexa Fluor 488 Antibody Labeling Kit, Invitrogen) for 1 h on ice. Single nuclei were separated from doublets, and higher-order nuclear aggregates using FSC and SSC as previously described (Spalding et al., 2005a). The purity of the sorted nuclei was documented by reanalyzing the sorted (NeuN−, NeuN+) populations (see Supplementary Figure 6). The nuclei were collected through centrifugation at 500 × g for 10 min and further processed for ^15^N measurements. Flow cytometry sorting was performed using the BD FACSAriaTM III.

### 2.6. Histopathological analysis

Human tissue samples underwent standard neuropathological work-up. For patient 1 histomorphological analysis included immunohistochemistry, isocitrate dehydrogenase 1 (IDH-1) wildtype analysis and IDH1 immunostaining as well as Ki67 immunolabeling. In patient 2 NeuN immunohistochemistry was performed and analyzed within the hippocampal specimen for neuronal cell densities within the pyramidal and granule cell layers.

### 2.7. Sample preparation for multi-isotope imaging mass spectrometry analysis

Cells that had incorporated ^15^N-thymidine were identified by multi-isotope imaging mass spectrometry (MIMS). For imaging, mass spectrometry samples underwent additional fixation with osmium tetroxide (Sigma-Aldrich, Germany) for 1.5 h at RT and were then embedded in araldite EPON resin (Sigma-Aldrich, Germany). Prior to analysis, samples were sliced into sections of 200 nm thickness using an ultramicrotome from Leica Microsystems (EM UC6). Samples were mounted on silicon wafers (Siegert Wafer GmbH, Aachen) for analysis. Representative semi-thin slices of 500 nm thickness were additionally mounted on microscope slides and stained with 1% toluidine blue to identify areas of interest using a light microscope.

In MIMS, a primary Cs + beam is scanned over the surface of the specimen to be analyzed. This induces the formation of secondary ions, which are released from the specimen, then are separated in a mass spectrometer according to their masses, and are detected by highly sensitive mass spectrometers before parallel ion counting for 5 to 7 ion species using electron multipliers. The sensitivity of the mass resolution of the instrument is sufficient to differentiate isotopes from each other, as ^15^N and ^14^N (measured in the form of negative CN ions, CN−; Nunez et al., 2017; Bonnin and Rizzoli, 2020). MIMS analyses for all samples were performed using a NanoSIMS 50 L instrument (Cameca, France). For tumor and hippocampus samples, secondary ions were generated using a primary current of ~30 pA (primary aperture D1 = 2). Prior to each measurement, an implantation of Cs + ions was performed at high current (L1 = 20,000, D1 = 1) on an area larger than the raster size used for analysis until steady state conditions were achieved.

Low resolution images were acquired at a raster size of either 90 × 90 μm (tumor sample) or 70 × 70 μm (hippocampus sample). To create the full image, images were taken from adjacent locations and stitched together in post-processing. To facilitate accurate stitching, an overlap of at least 5 μm between images was maintained in all directions. Two-dimensional images were obtained from single planes. High resolution images were obtained using a raster size of 12 × 12 μm and 40 × 40 μm, respectively, from locations that appeared high in ^15^N/^14^N in the low resolution images (tumor samples) or from random locations that had previously been analyzed (hippocampus samples). Three images (Berdugo-Vega et al., 2020) were obtained at each location.

All images were taken at a resolution of 256 × 256 pixels, with 5,000 cts/px, leading to an estimated resolution of ~78 nm/px in the high-resolution images, ~273 nm/px in the hippocampus low-resolution images, and ~350 nm/px in the tumor sample low-resolution images. The following masses were collected for each run: 12C14N (referred to as ^14^N in this report), 12C15N (referred to as ^15^N in this report), and 31P and 32S. The 32S peak was used to mark the location of cellular structures. Histology images were obtained using an Axio Imager M2 upright microscope (Zeiss, Germany). Samples for histology imaging were cut in thicker slices than NanoSIMS samples, and were stained with toluidine Blue (Merck, Germany) prior to imaging, according to the manufacturer’s instructions.

### 2.8. Image analysis and processing

The MIMS analysis technique collects chemical information from across the entire analysis area, creating an image where each pixel contains the mass spectra of all secondary ions collected at each particular location. However, in order to fully examine the MIMS data from each of these samples, it was first necessary to convert each image into a text file, so that the intensity of each pixel could be easily retrieved. This allows for mathematical operations and statistical tests to be performed on the image as a whole. It also allows the image to be examined on image processing software such as FIJI/ImageJ (NIH, Bethesda, MD, United States) and for the easy examination of regions of interest (ROIs). A more in-depth discussion on MIMS image generation and an overview on current practices of MIMS image have been published previously (Lechene et al., 2006).

In this study, MIMS images were processed using a custom Matlab script (the Mathworks Inc., Natick, MA, United States), adapted partially from the previous analysis scripts (Saka et al., 2014; Bonnin et al., 2021). In cases where more than one image was taken at a given location (such as in the high-resolution images), the intensities of each component image were summed together into a single image matrix. The ^15^N/^14^N ratio images were then obtained by dividing each data point in the numerator isotope with each data point in the denominator isotope. The resulting matrices were saved as text files. Images were then generated using FIJI/ImageJ (NIH, Bethesda, MD, United States). In the case of the low-resolution images, where multiple images were used to examine a large area, images were stitched together manually by aligning each image along overlapping regions. Average isotope ratios for individual cells in the tumor region were generated using FIJI/ImageJ. ROIs were drawn around each cell nucleus visible in the 14 N images by first creating a threshold image that filters out background, and then applying the Analyze Particle tool (green circles, Supplementary Figure 7). All ROIs were then saved in FIJI/ImageJ and applied to the appropriate ratio image for each experiment. Mean isotope ratios for each individual cell and cell region were generated using the Measure tool of FIJI/ImageJ (Schindelin et al., 2012). Cells with an average ^15^N/^14^N ratio > 10% higher than natural abundance were flagged as cells showing positive ^15^N-enrichment.

## 3. Results

### 3.1. Verification of the present protocol of *in vivo* ^15^N-thymidine labeling of adult humans followed by MIMS analysis

**Figure 1:**
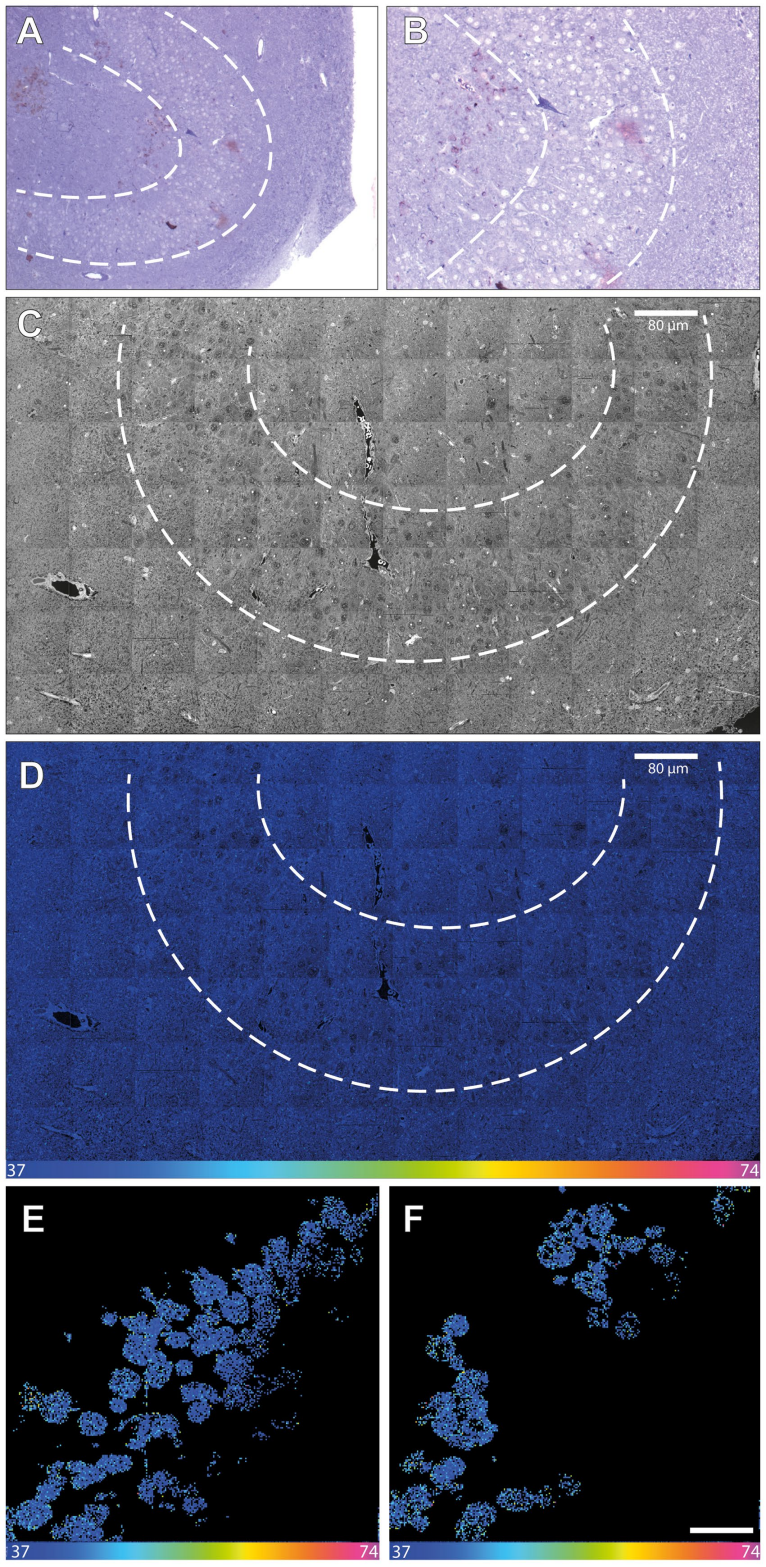
NanoSIMS analysis of human leucocytes after infusion with ^15^N-thymidine. **(A)** Light microscopic image of toluidine blue-stained leukocytes from patient 1 embedded in agarose and araldite resin after infusion with ^15^N-thymidine over a course of 24 h (100× magnification). **(B)** Higher magnification of a section of **(A)** (400×). **(C)** 31P MIMS image of a 200-nm section of the leukocytes depicted in **(A,B)**, identifying the nuclear chromatin used for the single cell-based identification of the region of interest (ROI) for subsequent ^15^N/^14^N quantification. **(D)** Mosaic ^15^N/^14^N MIMS hyperspectral image (HSI) of leukocytes obtained from patient 1 after infusion with ^15^N-thymidine for 24 h. The white box shows a leukocyte with increased nuclear ^15^N/^14^N, shown in panel **(G)** at higher magnification. **(E)** 14 N MIMS high-resolution image of leukocytes from patient 1 after infusion with ^15^N-thymidine for 24 h, including the leukocyte shown in the box in **(D)**. **(F)** Corresponding 31P MIMS image of the leukocytes identifying the nuclear chromatin. **(G)** Corresponding ^15^N/^14^N HSI of the leukocytes identifying a leukocyte with nuclear ^15^N accumulation (arrow). **(H)** ^14^N MIMS high-resolution image of leukocytes from patient 2 after infusion with ^15^N-thymidin for 24 h. **(I)** Corresponding 31P MIMS image of the leukocytes identifying the nuclear chromatin. **(J)** Corresponding ^15^N/^14^N HSI image of the leukocytes identifying a positive leukocyte with nuclear ^15^N accumulation (arrow) and a leukocyte with baseline ^15^N/^14^N ratio (arrowhead). Scale bar at bottom **(D,G,J)**: ^15^N/^14^N signal intensity increasing from natural ratio (37/10,000, left) to 2-fold (74/10,000, right).

Two adult human patients, a glioblastoma patient (referred to as patient 1) and a patient with epilepsy (referred to as patient 2, Supplementary Table 1; Supplementary Figures 1–4) were subjected, without any clinical harm or ethical concerns, to infusion with a total of 250 mg ^15^N-thymidine each over a period of 24 h (for details, see Materials and methods). We first verified that the present protocol of *in vivo* ^15^N-thymidine labeling of adult humans followed by MIMS analysis yielded similar results as previously reported in the seminal study by Steinhauser et al. (2012) and Senyo et al. (2013), that is, that the administered dose of ^15^N-thymidine was sufficient for MIMS detection of DNA synthesis in humans. To this end, we analyzed blood samples obtained on postoperative day 22 from patient 1 (i.e., 23 days after the end of ^15^N-thymidine infusion) and at day 54 post-infusionem from patient 2. We found 4 ^15^N-positive leukocyte nuclei out of 2,031 leukocytes analyzed (0.2%) in patient 1 (Figure 1), and 6 out of 2,225 (0.27%) in patient 2 (Figure 1). These data confirm previous evidence reported in the first *in vivo* ^15^N-thymidine labeling study of humans (Steinhauser et al., 2012; Senyo et al., 2013)and demonstrate the appropriateness of the present protocol to search for the generation of new cells in the adult human brain.

### 3.2. ^15^N-thymidine–positive cells in glioblastoma tissue demonstrate the ability to detect the generation of new cells in the human CNS by MIMS analysis

**Figure 2:**
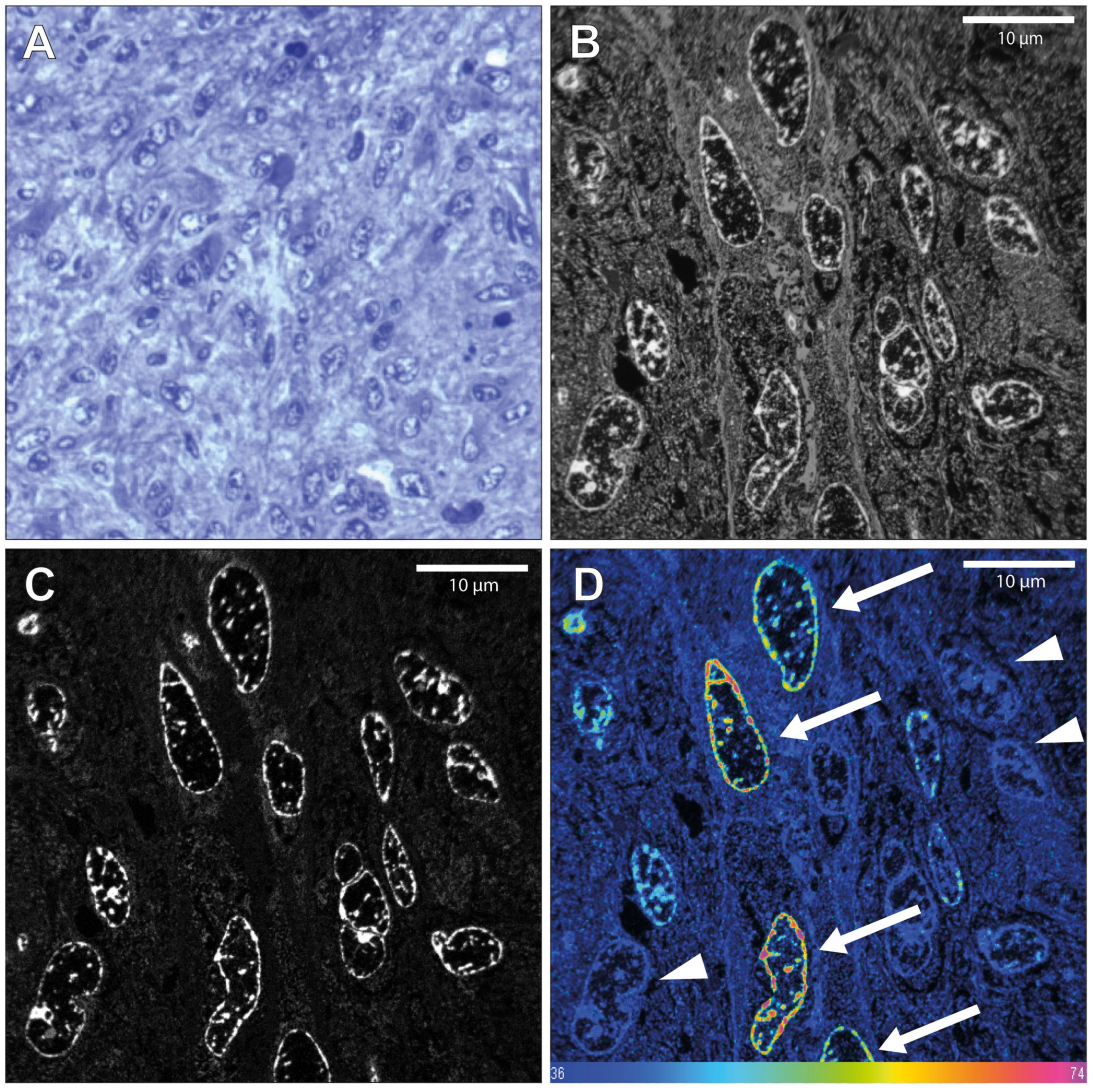
NanoSIMS analysis of glioblastoma tissue after infusion with ^15^N-thymidine. **(A)** Light microscopic image of a toluidine blue-stained 500 nm section of the glioblastoma tissue resected from patient 1, 12 h after the end of the infusion with ^15^N-thymidine for 24 h, embedded in araldite EPON resin. **(B)** 200 nm section 14 N MIMS image of the glioblastoma tissue after ^15^N-thymidine infusion. **(C)** Corresponding 31P MIMS image of the glioblastoma tissue after ^15^N-Table. ^15^N/^14^N MIMS HSI of the glioblastoma tissue after ^15^N-thymidine infusion. The arrows show glioblastoma cells with increased ^15^N/^14^N ratio, indicative of DNA synthesis during the labeling period. The arrowheads show cells with baseline ^15^N/^14^N ratio. Scale bar at bottom **(D)**: ^15^N/^14^N signal intensity increasing from natural ratio (37/10,000, left) to 2-fold (74/10,000, right).

Next, we analyzed a glioblastoma tissue sample, known to exhibit high proliferative activity that had been resected from patient 1 directly after the end of the ^15^N-thymidine infusion. ^15^N-positive neural cells were easily identified (7 out of 129, 5.43%, Figure 2). The mean ^15^N/^14^N ratios of these positive tumor cells ranged from 0.00406 (10.3% higher than the natural abundance of 0.00368) to 0.0054 (46.7% higher than natural abundance). This range is consistent with the notion that cells that underwent full S-phase during the 24 h *in vivo* labeling period, cells that underwent S-phase only partially, as well as cells that had been generated from the former cells but had themselves not undergone S-phase, were detected (Figure 2). These data demonstrate that the present protocol of *in vivo* ^15^N-thymidine labeling of adult humans followed by MIMS analysis is appropriate to detect the generation of new cells in the human CNS.

### 3.3. No evidence of ^15^N-thymidine–positive cells upon limited MIMS analysis of adult human hippocampus

**Figure 3:**
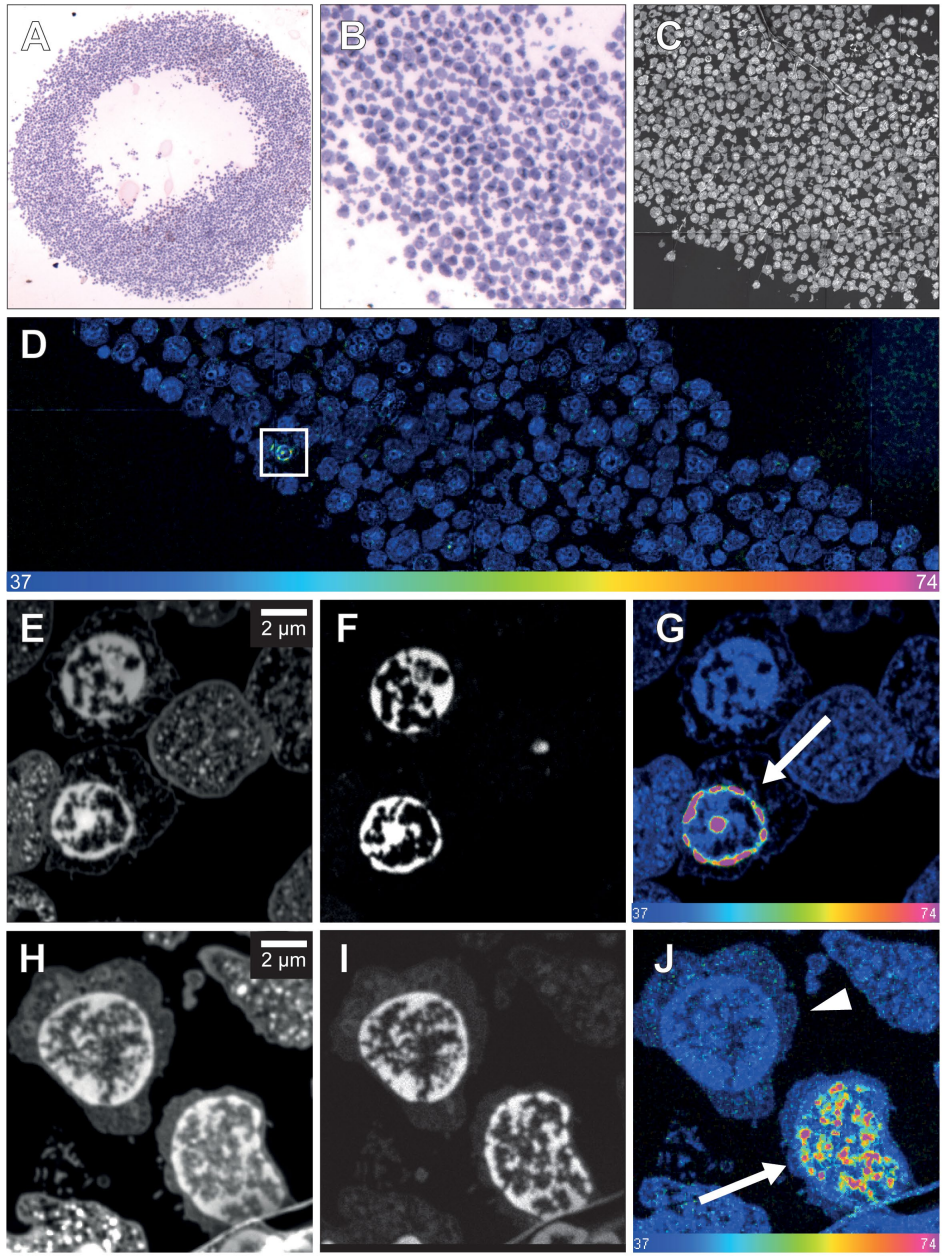
NanoSIMS analysis of adult human hippocampus tissue after infusion with ^15^N-thymidine. **(A)** Light microscopic image (10×) of a toluidine blue-stained 500-nmsection of the microdissected adult human dentate gyrus (between the dashed lines) after infusion with ^15^N-thymidine over a course of 24 h, followed by surgical resection of the hippocampus 43 days later. **(B)** Higher magnification (20×) of the image in **(A)**. **(C)** 200 nm section 14 N MIMS image of the adult human dentate gyrus (between the dashed lines) after labeling with ^15^N-thymidine for 24 h. **(D)** ^15^N/^14^N MIMS HSI the adult human dentate gyrus after labeling with ^15^N-thymidine for 24 h. All cells analyzed show baseline ^15^N/^14^N ratios, consistent with no detectable DNA synthesis during the labeling period. Note, that the direction and the size of the granular cell band of the dentate gyrus varies among the light microscopic **(A,B)** and the MIMS images **(C,D)**. **(E,F)** Two cutout examples of mosaic ^15^N/^14^N HSI image of NeuN-positive FAC-sorted hippocampal cells after infusion with ^15^N-thymidin over a course of 24 h (scare bar in panel F corresponds to 10 μm). In total, 1,198 cells were analyzed showing all baseline ^15^N/^14^N ratios, consistent with no detectable DNA synthesis. Scale bar at bottom **(D–F)**: ^15^N/^14^N signal intensity increasing from natural ratio (37/10,000, left) to 2-fold (74/10,000, right).

In light of these data, we explored whether the present protocol of *in vivo* ^15^N-thymidine labeling would suffice to detect the generation of new cells – if it occurs to any significant extent—in the adult human hippocampus by MIMS analysis. To this end, we used hippocampal tissue of patient 2, who suffered from a pharmaco-refractory focal temporal epilepsy and had undergone right-sided amygdalo-hippocampectomy 43 days after the end of the ^15^N-thymidine infusion. However, MIMS analysis of a very limited number of cells in the dentate gyrus dissected from the resected hippocampus tissue yielded negative results (Figure 3; Supplementary Figure 5). Specifically, of the 500 neurons in the granule cell layer analyzed in two 200-nm sections, none was positive for ^15^N-thymidine uptake.

As an alternative approach to detect newly generated hippocampal neurons, neuronal nuclei from a part of the hippocampal sample were first immunostained for NeuN and then enriched by FACS. A total of 1,198 neuronal nuclei were subsequently subjected to MIMS analysis (Figure 3; Supplementary Figure 6). However, again, none of these neuronal nuclei analyzed showed a positive ^15^N/^14^N signal.

## 4. Discussion

A seminal paper by Guo-li Ming, Hongjun Song and colleagues last year (Zhou et al., 2022) has provided support for a consensus view regarding whether and to what extent new neurons are being generated in the adult human hippocampus. According to that study, there is a massive decline in the generation of new dentate granule cells in the adult human hippocampus, down to less than 5% of that observed at the early child stage. Yet, the generation of a small number of newborn dentate granule cells can be observed in the adult human hippocampus. If their number is deduced from the number of the various types of progenitor cells, it is thought to amount to about 500–1,200 dentate granule cells being generated per day for one adult hippocampus (Zhou et al., 2022). This number is in the same range as that suggested in a landmark paper by Jonas Frisén and colleagues using retrospective 14C birthdating (about 600–700 newborn neurons per day; Spalding et al., 2013).

The key perspective of the present study is that we report a novel approach that should allow, in the future, to verify, or modify these numbers. In contrast to previous approaches that involved, for example, EdU labeling of resected hippocampal tissue in slice culture *in vitro* (Zhou et al., 2022), the present approach of ^15^N-thymidine labeling of adult humans followed by MIMS analysis constitutes a true *in vivo* labeling approach that is distinct from previous approaches. Our data with glioblastoma tissue resected from an adult human patient demonstrate that mitotically active cells and their progeny can be detected by this approach. In the case of the hippocampal tissue resected from an epilepsy patient, the lack of detection of ^15^N-thymidine–labeled neurons in the granule cell layer among the 500 neurons analyzed in two 200-nm sections was to be expected if the rate of generation of newborn neurons in the adult human dentate gyrus is indeed as low as reported (Knoth et al., 2010; Spalding et al., 2013; Bergmann et al., 2015; Dennis et al., 2017; Cipriani et al., 2018; Sorrells et al., 2018). Such a low probability poses a great challenge for the “standard” approach presented in Figure 3, in light of the sophisticated nature of MIMS analysis, including the duration of image acquisition, and the number of cells that can be analyzed in a single scan (see Materials and methods and Supplementary Figure 7). However, our continuative approach (Figure 3) to enrich neuronal nuclei after NeuN immunostaining by FACS prior to MIMS analysis provides a doable future perspective, as this approach can be scaled up. This should make it possible to obtain a sufficiently high number of hippocampal neuronal nuclei to allow the detection, and quantification, of ^15^N-thymidine–labeled neuronal nuclei, even at the reported low rate of generation of newborn neurons in the adult human dentate gyrus.

The lack of detection of a single ^15^N-thymidine–labeled neuron among the approximately 1,700 neurons analyzed here after a 24-h ^15^N-thymidine infusion implies that the rate of neurogenesis per year for the ≈10 million mature hippocampal neurons in total (Simic et al., 1997) must be less than 21.5% [i.e., <1 positive neuron (out of 1,700 analyzed neurons) * 5,900 (if analyzed all 10 million neuron) * 365 (if infusion had lasted for 1 year instead of 1 day) *100 (to result as an percentage)/10,000,000 neurons of the entire hippocampus ≤ 21.5%]. However, if we take the above-mentioned lowest number of 500 dentate granule cells being generated per day per hippocampus (Zhou et al., 2022), this would translate into a yearly rate of neurogenesis for the ≈10 million mature neurons in the adult human dentate gyrus of only 1.825% [500 neurons per day * 365 days * 100 (percent)/10,000,000 neurons = 182,500 new neurons per year]. Furthermore, if we assume a thickness of the adult human dentate gyrus of approximately 1.5 cm, 75,000 sections of 200-nm thickness would need to be analyzed by MIMS to cover the entire dentate gyrus. These calculations should be interpreted with caution: though the adult mammalian brain contains two neurogenic regions, there are distinct differences with regard to whether cell are replaced, i.e., turning over, vs. there is an additive process. These mechanisms may undermine the validity of estimating a neurogenesis rate, which further appears difficult to establish in light of an age-dependent decline in neurogenesis (Ninkovic et al., 2007; Rutten et al., 2007; Imayoshi et al., 2008; Appleby et al., 2011; Bergmann et al., 2012). Of note, using the FACS approach with subsequent MIMS analysis of sufficiently high numbers of neurons should narrow down the 21.5% stated above.

Additional aspects emerge from the data. One the one hand, potential limitations of the present data should be considered. The 24-h period of ^15^N-thymidine infusion we used might be insufficient to capture quiescent neural stem cells. Second, it should be noted that epilepsy, the major reason to resect hippocampal tissue from living adult humans, is known to lower, *per se*, the rate of hippocampal neurogenesis (Ammothumkandy et al., 2022). Hence, in the future, one should consider obtaining hippocampal tissue from ^15^N-thymidine-labeled healthy individuals who deceased within a time window after the end of labeling suitable for MIMS analysis. On the other hand, analogous to our approach for enrichment of neurons by FACS using NeuN antibody, one could perform an enrichment of human oligodendrocyte precursor cells by FACS using an appropriate antibody such as one against OLIG2. This would be an approach to determine the ^15^N-thymidine incorporation into these cells and hence their cell turnover, a topic of clinical relevance (Matsumoto et al., 2011; Zhang et al., 2020). With regard to studying cell turnover in cancer patients, a larger study in the future involving more individuals would be valuable. Finally, as an alternative to the various approaches discussed in this study to detect neurogenesis in the adult human brain, the application of magnetic resonance spectroscopy of the living human brain has been proposed (Manganas et al., 2007). However, there has been substantial critique regarding the validity of this approach (Friedman, 2008; Hoch et al., 2008).

We therefore suggest that pursuing the approach of *in vivo* ^15^N-thymidine labeling of adult humans followed by MIMS analysis described here, together with the scaling-up measures mentioned above, provides a promising future perspective to determine the rate of generation of newborn neurons in the adult human brain. On a more general note: the *in vivo* labeling of humans with ^15^N-thymidine followed by MIMS analysis of brain tissue constitutes a novel approach to study mitotically active cells and their progeny in the brain, and thus allows a broad spectrum of studies of brain physiology and pathology, including microcephaly.

## Data availability statement

The raw data supporting the conclusions of this article will be made available by the authors, without undue reservation.

## Ethics statement

The studies involving humans were approved by Ethikkommission der Medizinischen Fakultät, Universitätsklinikum Erlangen, Deutschland. The studies were conducted in accordance with the local legislation and institutional requirements. The participants provided nformed consent to participate in this study.

## Author contributions

SR: Data curation, Formal Analysis, Investigation, Methodology, Project administration, Writing – original draft. EB: Formal Analysis, Investigation, Methodology, Software, Writing – review & editing. T-DW: Formal Analysis, Investigation, Methodology, Software, Writing – original draft. J-LG-K: Conceptualization, Formal Analysis, Investigation, Supervision, Validation, Writing – original draft. SJ: Formal Analysis, Investigation, Methodology, Writing – review & editing. SB: Investigation, Methodology, Project administration, Writing – review & editing. IE: Investigation, Methodology, Project administration, Writing – review & editing. SG: Data curation, Investigation, Validation, Writing – review & editing. HH: Investigation, Supervision, Validation, Writing – review & editing. SG: Conceptualization, Methodology, Project administration, Writing – review & editing. TD: Conceptualization, Supervision, Writing – review & editing. CR: Supervision, Validation, Writing – review & editing. EE: Investigation, Methodology, Validation, Writing – review & editing. RH-B: Investigation, Methodology, Writing – review & editing. TB: Investigation, Methodology, Writing – review & editing. CD: Investigation, Methodology, Validation, Writing – review & editing. KA: Investigation, Methodology, Supervision, Writing – review & editing. US-S: Investigation, Methodology, Supervision, Writing – review & editing. AT: Conceptualization, Resources, Supervision, Validation, Writing – original draft. KR: Formal Analysis, Investigation, Methodology, Writing – original draft. SS: Conceptualization, Funding acquisition, Project administration, Resources, Supervision, Writing – review & editing. OB: Conceptualization, Data curation, Formal Analysis, Funding acquisition, Investigation, Writing – original draft. SR: Conceptualization, Formal Analysis, Investigation, Resources, Supervision, Writing – original draft. HH: Supervision, Validation, Writing – original draft, Conceptualization, Formal Analysis, Funding acquisition, Investigation.
