# Supplementary material for: Tracking cell turnover in human brain using 15N-thymidine imaging mass spectrometry
Source: Front Neurosci. 2023 Sep 28;17:1274607. doi: 10.3389/fnins.2023.1274607 (PMC10585107; doi:10.3389/fnins.2023.1274607)
Supplement: Supplementary file 1 [file Table_1.docx]

Supplementary Material

**Title**: Tracking cell turnover in human brain using 15N-thymidine imaging mass spectrometry

**Supplementary Table 1.** Patient characteristics

**Supplementary Figure 1.** Clinical images of patient 1

**Supplementary Figure 2.** Histological images of patient 1

**Supplementary Figure 3.** Clinical images of patient 2

**Supplementary Figure 4.** Histological images of patient 2

**Supplementary Figure 5.** Microdissection of the resected hippocampus

**Supplementary Figure 6.** FACS sorting of NeuN+ and NeuN- neurons

**Supplementary Figure 7.** ROI generation for ^15^N/^14^N quantification

**Supplementary Table 1.** Patient characteristics.

| *Pat-ID* | *Year of birth* | *Sex* | *Age* | *Prior medical history* | *Prior Medication* | *Family medical history* |
| --- | --- | --- | --- | --- | --- | --- |
| 1 | 1953 | female | 66 | Hypertension | Antihypertensives | No history of neuro-psychiatric diseases |
| 2 | 1992 | female | 27 | - Right temporal lobe epilepsy (first presentation in 1997) - Duane syndrome - Migraine - Contact dermatitis | Zonisamide, Eslicarabzepine, Folate.  Contraception using intrauterine device | Father suffering from epilepsy |

**Supplementary Figure 1.** Clinical images of patient 1


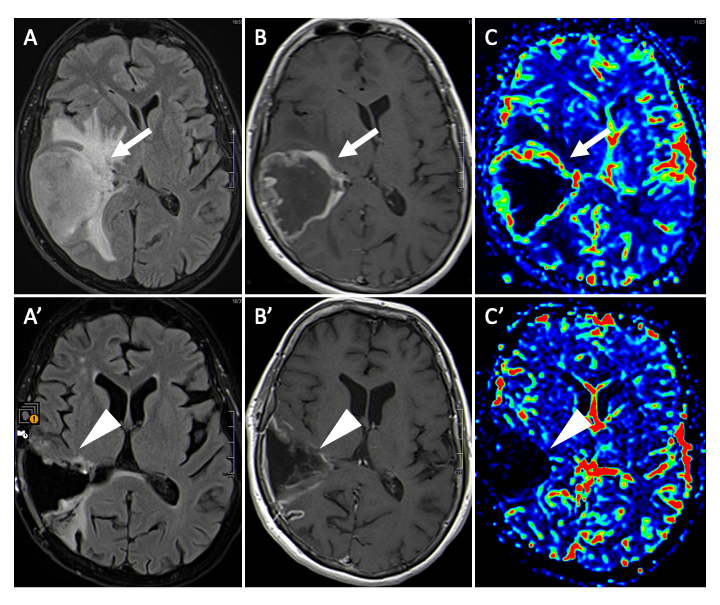


**A** Transverse MRI slice (T2-FLAIR) of patient 1 at the time of presentation to the hospital. The arrow shows a right-sided temporo-parietal mass with hyperintense T2 signal and surrounding T2 hyperintense edema. **B** Corresponding T1 MRI slice to A after administration of gadolinium contrast agent. Contrast enhancement is found in the solid peripheral tumor parts with T1-C+ hypointense central signal representing tumor necrosis. **C** Corresponding perfusion-weighted MRI slice to A. The arrow shows hyperperfusion in the solid peripheral tumor parts. **A’** Post-operative transverse MRI slice (T2-FLAIR) at the same level as A. The arrow head shows the T2 hypointense tumor cavity after surgical resection of the mass. Hyperintense peripheral edema can still be found surrounding the resection site. **B’** Corresponding T1-C+ MRI slice to A agent showing removal of most C+ malignant tissue. **C’** Perfusion-weighted MR imaging shows absence of hyperperfused tissue after surgery.

**Supplementary Figure 2.** Histological images of patient 1


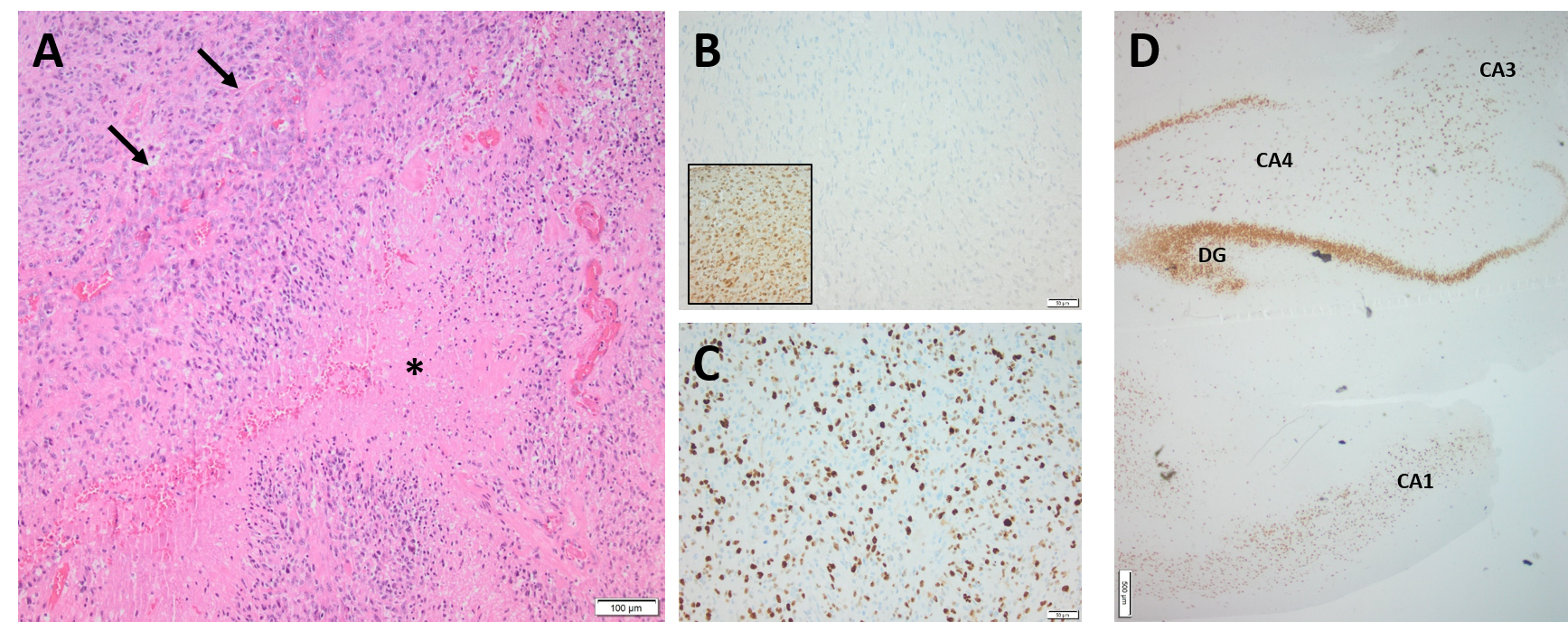


**A** HE staining shows typical histomorphological features of a Glioblastoma (CNS WHO Grade 4): a malignant, pleomorphic glial tumor with microvacular proliferation (black arrows) and palisading necrosis (asterisk). **B** Immunohistochemistry confirmed IDH-1 wildtype (R132H); inset in B: on slide positive control for IDH1 immunostaining. **C** Ki67 immunolabeling highlights the high proliferative activity of the tumor sample.

**Supplementary Figure 3.** Clinical images of patient 2


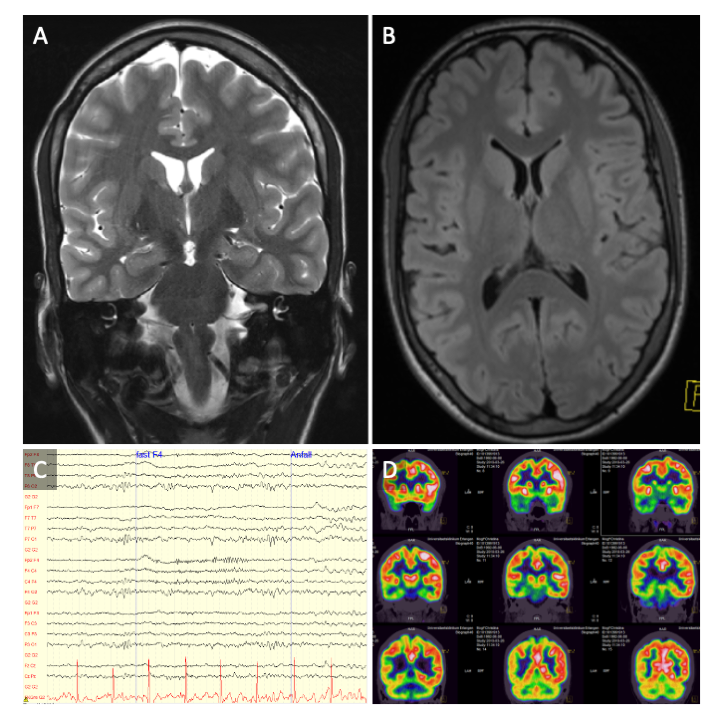


Coronal T2-weighted (**A**) and transverse T2-FLAIR-weighted (**B**) MRI slices show absence of morphological abnormalities of the amygdalohippocampal area in patient 2. **C** Representative EEG recording of patient 2 at the time of a seizure. **D** ^18^F-FDG PET scan reveals a glucose hypometabolism in the right temporal lobe.

**Supplementary Figure 4.** Histological images of patient 2
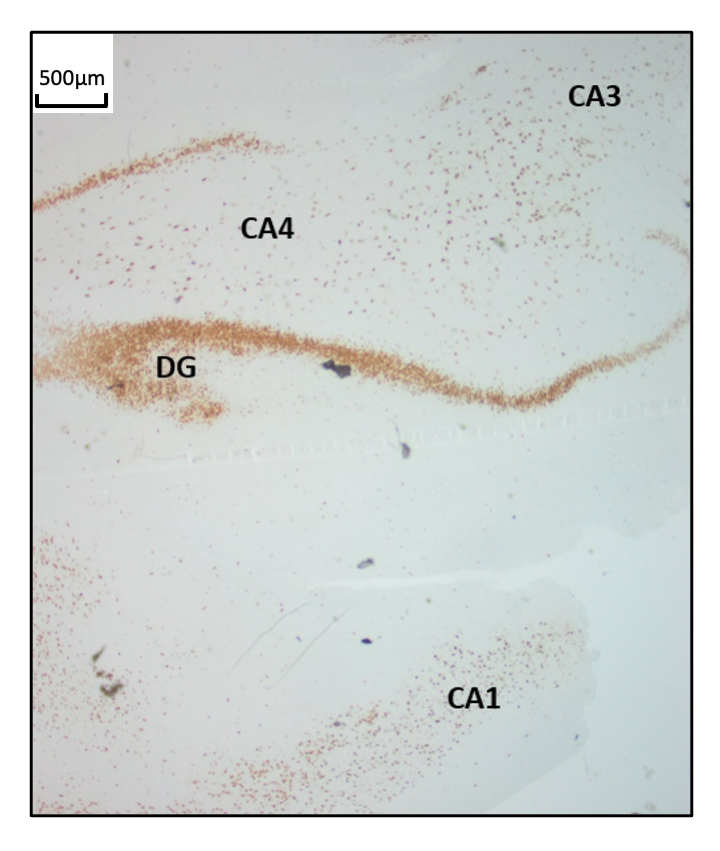


NeuN immunohistochemistry performed in the hippocampal of patient 2 reveals regular neuronal cell densities within the pyramidal cell layer (CA1, CA3 and CA4; sector CA2 is not visible due to surgical artifacts). The granule cell layer of the dentate gyrus (DG) shows no dispersion or cell loss.


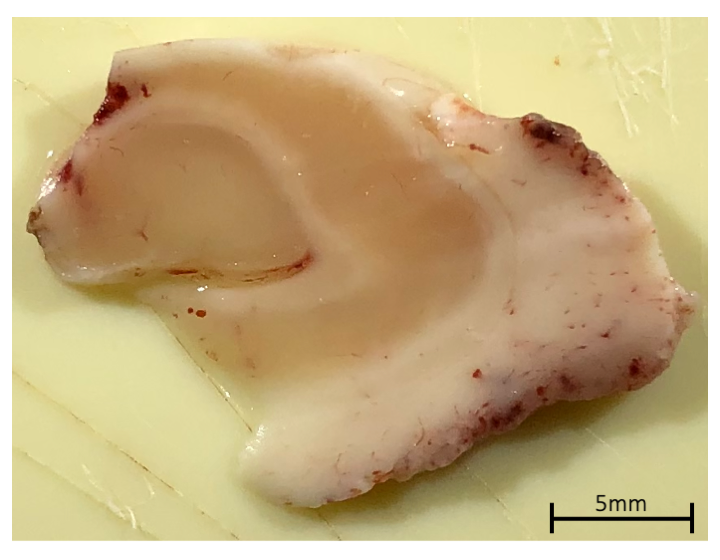
**Supplementary Figure 5.** Resected hippocampus prior to microdissection.

Macroscopic image of a coronal section of the surgically resected right hippocampus of patient 2. This section was used for microdissection of the specimen into small consecutive cubes for subsequent embedding and NanoSIMS analysis.

**Supplementary Figure 6.** FACS sorting of NeuN+ hippocampal neurons


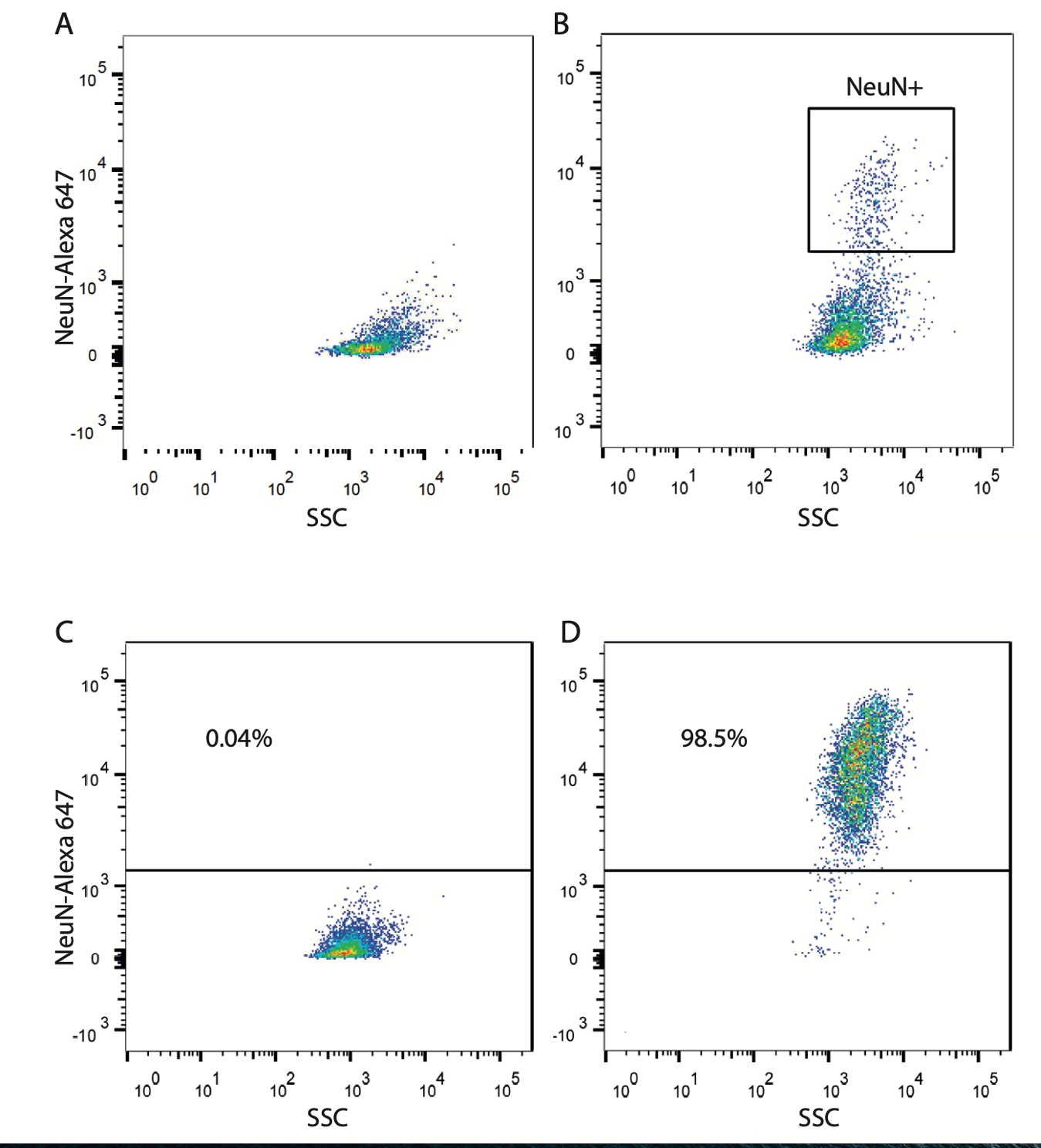


**A** Incubation of isolated hippocampal nuclei with isotype control IgG **B** Staining of hippocampal nuclei with directly conjugated NeuN antibody **C, D** Reanalysis of the NeuN+ fraction (inset B) reveals a 98.5% purity of the sort.

**Supplementary Figure 7.** ROI generation for ^15^N/^14^N quantification


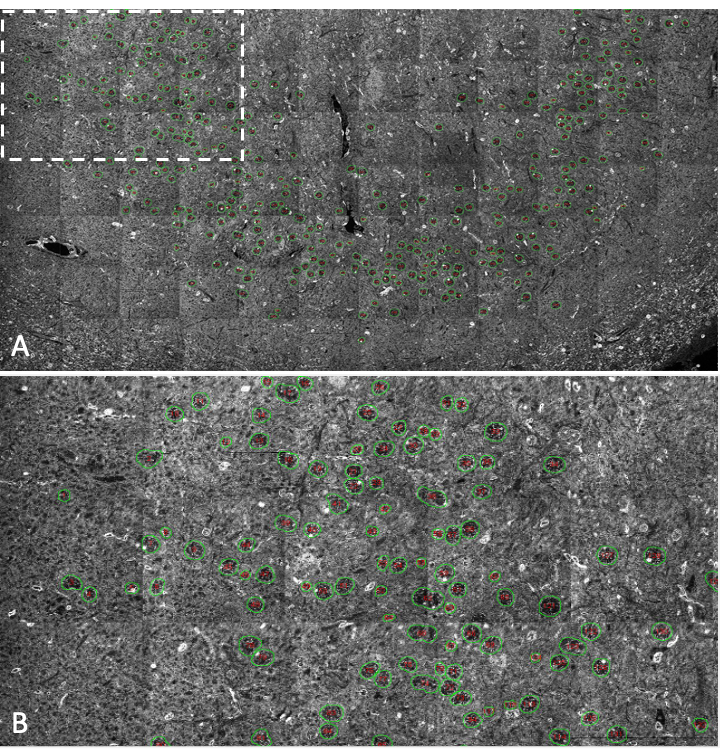


**A** ^14^N mosaic image of the dentate gyrus of patient 2 used for quantification of ^15^N/^14^N. ROIs were drawn around each cell nucleus visible in the P images as outlined in the methods sections. **B** Close-up of inset (A).
